# Genetic Predisposition to Increased Blood Cholesterol and Triglyceride Lipid Levels and Risk of Alzheimer Disease: A Mendelian Randomization Analysis

**DOI:** 10.1371/journal.pmed.1001713

**Published:** 2014-09-16

**Authors:** Petroula Proitsi, Michelle K. Lupton, Latha Velayudhan, Stephen Newhouse, Isabella Fogh, Magda Tsolaki, Makrina Daniilidou, Megan Pritchard, Iwona Kloszewska, Hilkka Soininen, Patrizia Mecocci, Bruno Vellas, for the Alzheimer's Disease Neuroimaging Initiative, Julie Williams, for the GERAD1 Consortium, Robert Stewart, Pak Sham, Simon Lovestone, John F. Powell

**Affiliations:** 1 King's College London, Institute of Psychiatry, Psychology and Neuroscience, London, United Kingdom; 2 Department of Psychiatry, State Key Laboratory of Brain and Cognitive Sciences, and Centre for Genomic Sciences, Li Ka Shing Faculty of Medicine, the University of Hong Kong, Hong Kong; 3 Neuroimaging Genetics, QIMR Berghofer Medical Research Institute, Herston, Queensland, Australia; 4 Department of Health Sciences, Psychiatry for the Elderly, University of Leicester, United Kingdom; 5 Memory and dementia center, 3rd Department of Neurology, Aristotle University of Thessaloniki, Greece; 6 Department of Old Age Psychiatry & Psychotic Disorders, Medical University of Lodz, Lodz, Poland; 7 Department of Neurology, Kuopio University Hospital and University of Eastern Finland, Kuopio, Finland; 8 Section of Gerontology and Geriatrics, Department of Medicine, University of Perugia, Perugia, Italy; 9 Department of Internal and Geriatrics Medicine, INSERM U 1027, Gerontopole, Hôpitaux de Toulouse, Toulouse, France; 10 MRC Centre for Neuropsychiatric Genetics and Genomics, Department of Psychological Medicine and Neurology, School of Medicine, Cardiff University, Cardiff, United Kingdom; 11 University of Oxford, Department of Psychiatry, Warneford Hospital, Oxford, United Kingdom; University of Bristol, United Kingdom

## Abstract

**Background:**

Although altered lipid metabolism has been extensively implicated in the pathogenesis of Alzheimer disease (AD) through cell biological, epidemiological, and genetic studies, the molecular mechanisms linking cholesterol and AD pathology are still not well understood and contradictory results have been reported. We have used a Mendelian randomization approach to dissect the causal nature of the association between circulating lipid levels and late onset AD (LOAD) and test the hypothesis that genetically raised lipid levels increase the risk of LOAD.

**Methods and Findings:**

We included 3,914 patients with LOAD, 1,675 older individuals without LOAD, and 4,989 individuals from the general population from six genome wide studies drawn from a white population (total *n* = 10,578). We constructed weighted genotype risk scores (GRSs) for four blood lipid phenotypes (high-density lipoprotein cholesterol [HDL-c], low-density lipoprotein cholesterol [LDL-c], triglycerides, and total cholesterol) using well-established SNPs in 157 loci for blood lipids reported by Willer and colleagues (2013). Both full GRSs using all SNPs associated with each trait at *p*<5×10^−8^ and trait specific scores using SNPs associated exclusively with each trait at *p*<5×10^−8^ were developed. We used logistic regression to investigate whether the GRSs were associated with LOAD in each study and results were combined together by meta-analysis. We found no association between any of the full GRSs and LOAD (meta-analysis results: odds ratio [OR] = 1.005, 95% CI 0.82–1.24, *p* = 0.962 per 1 unit increase in HDL-c; OR = 0.901, 95% CI 0.65–1.25, *p* = 0.530 per 1 unit increase in LDL-c; OR = 1.104, 95% CI 0.89–1.37, *p* = 0.362 per 1 unit increase in triglycerides; and OR = 0.954, 95% CI 0.76–1.21, *p* = 0.688 per 1 unit increase in total cholesterol). Results for the trait specific scores were similar; however, the trait specific scores explained much smaller phenotypic variance.

**Conclusions:**

Genetic predisposition to increased blood cholesterol and triglyceride lipid levels is not associated with elevated LOAD risk. The observed epidemiological associations between abnormal lipid levels and LOAD risk could therefore be attributed to the result of biological pleiotropy or could be secondary to LOAD. Limitations of this study include the small proportion of lipid variance explained by the GRS, biases in case-control ascertainment, and the limitations implicit to Mendelian randomization studies. Future studies should focus on larger LOAD datasets with longitudinal sampled peripheral lipid measures and other markers of lipid metabolism, which have been shown to be altered in LOAD.

*Please see later in the article for the Editors' Summary*

## Abstract 2

In this study, Proitsi and colleagues use a Mendelian randomization approach to dissect the causal nature of the association between circulating lipid levels and late onset Alzheimer's Disease (LOAD) and find that genetic predisposition to increased plasma cholesterol and triglyceride lipid levels is not associated with elevated LOAD risk.

*Please see later in the article for the Editors' Summary*

## Editors' Summary

**Background:**

Currently, about 44 million people worldwide have dementia, a group of brain disorders characterized by an irreversible decline in memory, communication, and other “cognitive” functions. Dementia mainly affects older people and, because people are living longer, experts estimate that more than 135 million people will have dementia by 2050. The commonest form of dementia is Alzheimer disease. In this type of dementia, protein clumps called plaques and neurofibrillary tangles form in the brain and cause its degeneration. The earliest sign of Alzheimer disease is usually increasing forgetfulness. As the disease progresses, affected individuals gradually lose their ability to deal with normal daily activities such as dressing. They may become anxious or aggressive or begin to wander. They may also eventually lose control of their bladder and of other physical functions. At present, there is no cure for Alzheimer disease although some of its symptoms can be managed with drugs. Most people with the disease are initially cared for at home by relatives and other unpaid carers, but many patients end their days in a care home or specialist nursing home.

**Why Was This Study Done?:**

Several lines of evidence suggest that lipid metabolism (how the body handles cholesterol and other fats) is altered in patients whose Alzheimer disease develops after the age of 60 years (late onset Alzheimer disease, LOAD). In particular, epidemiological studies (observational investigations that examine the patterns and causes of disease in populations) have found an association between high amounts of cholesterol in the blood in midlife and the risk of LOAD. However, observational studies cannot prove that abnormal lipid metabolism (dyslipidemia) causes LOAD. People with dyslipidemia may share other characteristics that cause both dyslipidemia and LOAD (confounding) or LOAD might actually cause dyslipidemia (reverse causation). Here, the researchers use “Mendelian randomization” to examine whether lifetime changes in lipid metabolism caused by genes have a causal impact on LOAD risk. In Mendelian randomization, causality is inferred from associations between genetic variants that mimic the effect of a modifiable risk factor and the outcome of interest. Because gene variants are inherited randomly, they are not prone to confounding and are free from reverse causation. So, if dyslipidemia causes LOAD, genetic variants that affect lipid metabolism should be associated with an altered risk of LOAD.

**What Did the Researchers Do and Find?:**

The researchers investigated whether genetic predisposition to raised lipid levels increased the risk of LOAD in 10,578 participants (3,914 patients with LOAD, 1,675 elderly people without LOAD, and 4,989 population controls) using data collected in six genome wide studies looking for gene variants associated with Alzheimer disease. The researchers constructed a genotype risk score (GRS) for each participant using genetic risk markers for four types of blood lipids on the basis of the presence of single nucleotide polymorphisms (SNPs, a type of gene variant) in their DNA. When the researchers used statistical methods to investigate the association between the GRS and LOAD among all the study participants, they found no association between the GRS and LOAD.

**What Do These Findings Mean?:**

These findings suggest that the genetic predisposition to raised blood levels of four types of lipid is not causally associated with LOAD risk. The accuracy of this finding may be affected by several limitations of this study, including the small proportion of lipid variance explained by the GRS and the validity of several assumptions that underlie all Mendelian randomization studies. Moreover, because all the participants in this study were white, these findings may not apply to people of other ethnic backgrounds. Given their findings, the researchers suggest that the observed epidemiological associations between abnormal lipid levels in the blood and variation in lipid levels for reasons other than genetics, or to LOAD risk could be secondary to variation in lipid levels for reasons other than genetics, or to LOAD, a possibility that can be investigated by studying blood lipid levels and other markers of lipid metabolism over time in large groups of patients with LOAD. Importantly, however, these findings provide new information about the role of lipids in LOAD development that may eventually lead to new therapeutic and public-health interventions for Alzheimer disease.

**Additional Information:**

Please access these websites via the online version of this summary at http://dx.doi.org/10.1371/journal.pmed.1001713.

- The UK National Health Service Choices website provides information (including personal stories) about Alzheimer's disease
- The UK not-for-profit organization Alzheimer's Society provides information for patients and carers about dementia, including personal experiences of living with Alzheimer's disease
- The US not-for-profit organization Alzheimer's Association also provides information for patients and carers about dementia and personal stories about dementia
- Alzheimer's Disease International is the international federation of Alzheimer disease associations around the world; it provides links to individual associations, information about dementia, and links to World Alzheimer Reports
- MedlinePlus provides links to additional resources about Alzheimer's disease (in English and Spanish)
- Wikipedia has a page on Mendelian randomization (note: Wikipedia is a free online encyclopedia that anyone can edit; available in several languages)

## Introduction

Altered lipid metabolism has been extensively implicated in late onset Alzheimer disease (LOAD) pathogenesis but the molecular basis of this relationship is not well understood. Cell biological studies support a critical involvement of lipid raft cholesterol in the modulation of Aβ precursor protein processing by β-secretase and γ-secretase resulting in altered Aβ production (reviewed in [1]). In the brain, apolipoprotein E (APOE) acts as the major cholesterol transporter, taken up into neurones via low density lipoprotein receptor (LDLR) family members. APOE is lipidated by the cholesterol transporter ABCA1 in astrocytes and its correct lipidation is necessary for binding and clearance of Aβ from the brain [2]. Additionally, APOE is a crucial regulator of triglyceride metabolism throughout the body [3].

In addition to the APOE gene, many of the LOAD susceptibility loci identified through genome wide association (GWA) studies and meta-analyses are also involved in lipid metabolism [4]–[6]. For example, CLU, or APOJ, is the second main lipoprotein in the brain after APOE; PICALM and BIN1 are implicated in receptor mediated endocytosis; and ABCA7 is involved in the efflux of lipids from cells to lipoproteins.

Epidemiological studies have shown associations between high cholesterol levels in midlife and LOAD risk [7]–[9], and statins have been shown to have a protective effect against the development of dementia [10]–[12]. However contradictory results have also been reported, with other epidemiological studies reporting no association of lipid levels on LOAD risk [13],[14] or a decline in cholesterol levels before the onset of dementia [12],[15] and randomized control trials overall finding no benefit of statin treatment [16]–[19].

The aim of this study was to examine whether genetic predisposition to increased blood cholesterol and triglyceride levels (i.e., dyslipidemia) plays an aetiological role in LOAD. Consequently genetic risk variants, which affect lipid metabolism, would influence risk of LOAD through changes in lipid levels.

This is the first genetic study, to our knowledge, to investigate the causal nature of the relationship between lipid dysregulation and LOAD using such an approach, the results of which have the potential for public health interventions.

## Methods

Ethical approval was obtained for all cohorts in the corresponding centres.

**Figure 1:**
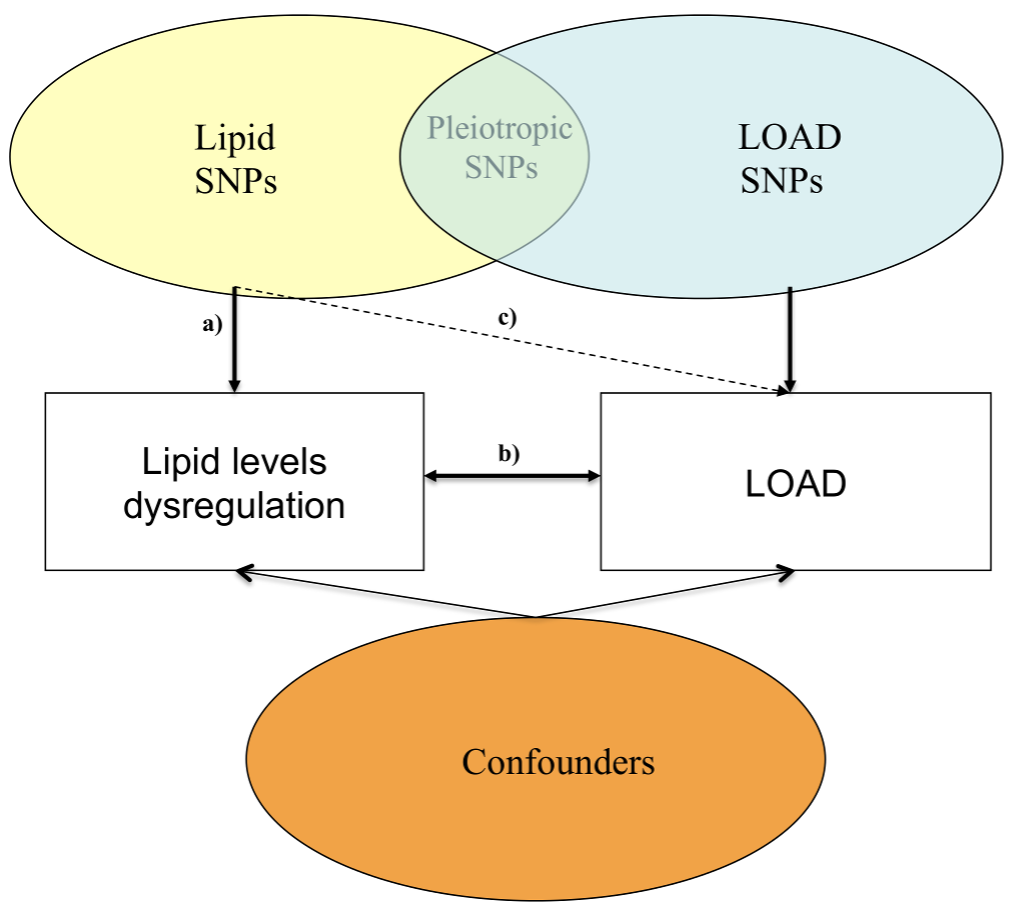
Possible mechanisms through which susceptibility genes act on lipid blood levels and LOAD. (a) Relationship between lipid SNPs and altered blood lipid levels; (b) relationship between altered blood lipid levels and LOAD; and (c) relationship between SNPs associated with altered lipid levels and LOAD.

We have employed a Mendelian randomization approach (Figure 1), which uses the principle that the random meiotic assortment of genotypes is independent of confounding non-genetic factors or disease processes.

Since the effects of individual loci identified through GWA studies and meta-analyses are small, we examined whether genotype risk scores (GRSs), based on the joint additive effect of 157 well-established independent loci involved in lipid metabolism (*p*<5×10^−8^) in a recent GWA study and meta-analysis by Willer and colleagues [20] of 188,577 participants, influence LOAD, in a sample of 10,578 participants, comprising 3,914 patients with LOAD (cases), 1,675 older individuals without LOAD, and 4,989 individuals from the general population. We analysed GRS for the phenotypes blood high-density lipoprotein cholesterol (HDL-c), plasma low-density lipoprotein cholesterol (LDL-c), total plasma cholesterol (TC), and plasma triglycerides (TG).

### Study Design and Participants

This study utilized data from participants from three independent study groups composed of six GWA studies. All individuals included in this study have provided written informed consent.

The first group was the Medical Research Council (MRC)-Wellcome Trust Case Control Consortium (WTCCC2) group including 3,292 individuals with LOAD (cases), 1,223 older individuals without LOAD (controls), and 5,074 individuals from the general population (population controls), consisting of four cohorts: 3,216 cases and 1,165 controls from the GERAD1 cohort (Genetic and Environmental Risk for Alzheimer's disease) consortium [4] genotyped on the Illumina 610-quad chip; 18 LOAD cases and ten controls from the MRC Brain cohort, genotyped on the Illumina 666W-Quad chip; and 5,074 population controls from the WTCCC2 publically available control cohorts (www.wtccc.org.uk/ccc2/: the 1958 British Birth Cohort [WTCCC2 1958 BC] and UK Blood Service Collection [WTCCC2 National Blood Donors (NBS)]), genotyped on the Illumina 1.2M chip.

The second group was the Institute of Psychiatry Plus (IOP+) group including 450 individuals who were cases, and 365 individuals who were controls, from the AddNeuroMed (ANM) cohort (362 cases, 237 controls) [21], and individuals from the Dementia Case Register (88 cases and 128 controls). These groups were genotyped on the Illumina 610-Quad chip in two different batches and merged together (batch 1: 222 cases, 111 controls; batch 2: 228 cases and 254 controls). Additionally, batch 1 contained 99 and batch 2 contained 78 individuals with mild cognitive impairment (MCI).

The third group consisted of 330 individuals who were cases and 187 who were controls obtained from the Alzheimer's Disease Neuroimaging Initiative (ADNI) database (adni.loni.usc.edu), genotyped on the Illumina 610-Quad chip.

Further details on all groups and cohorts are found in Text S1.

All individuals contributing data for this study were white. All individuals with LOAD (cases) met criteria for either probable (NINCDS-ADRDA, DSM-IV) or definite (CERAD) AD [22]. All non-population individuals who were controls were screened for dementia using the MMSE or ADAS-cog, or were determined to be free from dementia at neuropathological examination or had a Braak score ≤2.5. All individuals with LOAD (cases) had an age of onset ≥60 years and controls were ≥60 years at examination. The 58BC individuals (population controls) were around 54 years at the age of collection and no age was available for the NBS cohort. Because genotype data were used from multiple sources, stringent quality control (QC) filters were applied since differential genotyping error rates between groups could result in spurious associations when the data are combined [23],[24]. These filters were applied separately to each of these groups to remove poorly performing samples followed by SNP QC on each group. Prior to individual and SNP QC the MRC brain cohort was merged with the GERAD1 cohort after removing symmetric SNPs and flipping SNPs with opposite strands (from now on referred to as the MRC cohort).

### GWA Quality Control

Individual QC filters in the MRC, WTCCC2 58C, WTCCC2 NBS, IOP+, and ADNI datasets were applied using tools implemented in PLINK [25]. QC for the IOP+ group took place separately for the two different batches.

Briefly, we excluded individuals with (a) gender mismatches (M>0.8, male; F<0.2, female rule in PLINK; (b) an individual call rate ≤98%; (c) individuals with autosomal heterozygosity outside ±4 standard deviation (SD) of the mean heterozygosity; and (d) duplicates and cryptically related by calculating identity by descent (IBD) estimates for all possible pairs of individuals in PLINK and removing one of each pair with an IBD estimate ≥0.1875 (the level expected for second cousins). Each of the five datasets were then merged with genotypes from 210 unrelated European (CEU), Asian (CHB and JPT), and Yoruban (YRI) samples from the HapMap project (www.hapmap.org). Following removal of SNPs in extensive regions of linkage disequilibrium and pruning of SNPs if any pair within a 50-SNP window had r^2^>0.2, principal components analysis (PCA) as implemented in SMARTPCA [26] was used to infer continuous axes of genetic variation. Eigenvectors were calculated on the basis of the linkage disequilibrium (LD)-pruned subsets of each of the merged datasets to identify and then remove individuals of divergent ancestry displayed by plotting the first two principal components and using K-means clustering.

EIGENSOFTplus [27] was then applied to each of the datasets to additionally correct for population substructure, and genetic outliers defined as individuals whose ancestry is at least 6 SDs from the mean on one of the top ten axes of variation were removed. Four principal components explained most of the variation in the IOP+ and ADNI datasets and were extracted in order to be used as covariates in further analyses. Since the MRC and WTCCC2 datasets were merged at a later stage, extraction eigenvectors took place after sample merging.

### SNP Quality Control

Because of unresolved genotype-calling issues with a proportion of SNPs on the sex chromosomes in the 610 cohort, only autosomal SNPs were included in SNP QC for all cohorts. Briefly SNP QC took place (a) including SNPs with MAF>1%; (b) including SNPs with missingness <3% if MAF≥0.05 and SNPs with missingness <1% if MAF<0.05, and (c) excluding SNPs with HWE *p*≤1×10^−4^ in controls.

### Imputation

Since some of the SNPs to be used in this study were not included on the Illumina platform or failed QC, imputation took place using IMPUTE_2.2.2 [28] and the 1000G phase1 integrated reference panel (April 2012, National Center for Biotechnology Information [NCBI] build 37) (Text S1).

### Final QC Steps

The MRC dataset was then merged together with the WTCCC2 58C and WTCCC2 NBS datasets and the two IOP+ batches were also merged together. Symmetric SNPs were excluded and PLINK was used to identify incorrect strand assignment by utilizing LD patterns and exclude SNPs where the strand may have been incorrectly assigned between the three datasets. The merged datasets underwent an additional round of individual QC as described above and EIGENSOFTplus was applied to correct for population substructure as described. Four principal components explained most of the variation in the data and were extracted to be used as covariates in further analyses. Final QC resulted in 3,234 individuals with LOAD (cases), 1,175 individuals who were controls, and 4,989 individuals who were population controls from the MRC-WTCCC2 group (*n* = 9,398); 350 individuals with LOAD (cases) and 313 individuals who were controls from IOP+ group (*n* = 663); and 330 individuals with LOAD (cases) and 187 individuals who were controls from the ADNI group (*n* = 517), a total of 10,578 individuals included in all analyses.

### Outcomes

The main outcome of the study was LOAD status (dichotomous) in the 10,578 individuals included in analyses with available imputed data.

A total of 227 individuals with LOAD (cases) and 196 individuals who were controls of the ANM cohort of the IOP+ group had HDL-c, LDL-c, TC, and TG serum levels (mmol/l) available. Additionally, lipid serum data were also available for 127 individuals with MCI from the ANM cohort who had undergone GWA/imputation together with LOAD cases and controls of the ANM cohort.

### Genotype Risk Score Construction

Genotype scores were calculated for each of the four lipid phenotypes (TC, LDL-c, HDL-c, and TG) using the SNPs at 157 independent loci associated with plasma lipids at (*p*<5×10^−8^) as reported by Willer and colleagues [20]. To avoid any spurious associations the rs4420638 SNP within the APOE locus was excluded from further analyses and we used SNAP [29] to investigate linkage disequilibrium patterns between the blood lipid SNPs and SNPs associated with LOAD in the latest LOAD meta-analysis finding no significant linkage disequilibrium (r^2^<0.2). We also excluded SNP rs581080 in the TTC39B locus, SNP rs9411489 in the ABO locus, and SNP rs3177928 in the human leukocyte antigen (HLA) locus because they failed genotyping/imputation QC and we could not find any successfully genotyped/imputed SNPs with r^2^>0.8 to use as proxies. We initially constructed a GRS that included all SNPs associated with each target lipid trait at a pre-specified *p*-value threshold of *p*<5×10^−8^ (full score); we therefore used 69 HDL-c SNPs, 55 LDL-c SNPs, 40 TG SNPs, and 70 TC SNPs for the construction of the respective full score. Since one of the prerequisites for a Mendelian randomization study is that there must not be pleiotropic effects of the genetic variants of interest and since there is a considerable overlap of SNPs associated with each trait (Figure S1), a second score was constructed using SNPs exclusively associated with the target lipid trait at a *p*-value threshold of *p*<5×10^−8^ (trait specific score); we therefore used 45 HDL-c SNPs, nine LDL-c SNPs, 18 TG SNPs, and 18 TC SNPs for the construction of the respective trait specific score.

Full and trait specific risk scores (GRSs) were constructed in PLINK using the –score option [25] and assuming that each SNP in the panel acts independently and contributes to the risk of LOAD in an additive manner.

The weighted risk scores were constructed by multiplying each SNP by its relative effect size (β-coefficient) obtained from Willer and colleagues [20] and selecting as the “risk” allele that was associated with increased LDL-c, TG, and TC levels and decreased HDL-c levels.

We summed the products of each score and divided them by the number of non-missing SNPs genotyped/imputed for each individual, creating a score per non missing SNP. The GRSs were further standardized and results are expressed per 1 SD of each GRS.

Individuals missing ≥5% of the SNPs for each GRS were excluded. Table S1 presents the details of the SNPs used for the construction of the GRSs.

### Statistical Analyses

All analyses were performed in STATA 12 (Stata Statistical Software: Release 12, StataCorp LP). Power calculations were performed using QUANTO (http://hydra.usc.edu) to estimate the power of this study.

### Association of Serum Lipid Levels with Each Lipid Genotype Risk Score and LOAD Status

Serum lipid levels (mmol/l) available for the ANM subset were converted to mg/dl by multiplying HDL-c, LDL-c, and TC by 38.67 and TG by 88.57. Each lipid was regressed against age, age-squared, and gender, and the residuals were inverse normal transformed.

Linear regression analyses were performed using the inverse normal lipid traits values as the dependent variable and the respective GRS as the independent variable (first stage equations) using the 227 LOAD cases and 196 elderly controls from the ANM study. To increase power we also included the 127 individuals with MCI. These first stage equations were later used to weigh the association of each GRS with LOAD status. We also used logistic regression analyses to investigate the association of the inverse normal transformed lipid traits levels with LOAD status in the ANM cohort.

### Instrumental Variable Analyses

We used instrumental variable (IV) estimators to quantify the strength of the causal association between lipid traits and LOAD. We used two different procedures for our IV analysis.

#### Instrumental variable analysis using individual level data

For the main analysis of this study we investigated the association of each of the four lipid trait GRSs with LOAD. Logistic regression analyses were used to test for the association of each GRS with LOAD separately in each group adjusting for the first four PCS extracted during QC for each group. It was not possible to include a covariate for each chip for the MRC-WTCCC2 group as only population controls were genotyped on the 1.2 M chip. Similarly, it was not possible to include a covariate for each of the four cohorts of the MRC-WTCCC2 group, as the WTCCC2 groups included only population controls. The IOP+ cohort was adjusted for an additional covariate denoting genotyping batch. The instrumental variable estimate for each lipid trait was obtained by dividing the LOAD-GRS log OR estimate of each group (second stage equation) with the respective beta estimate of the linear regression of each lipid trait against the respective GRS (lipid trait-GRS) from the 550 individuals of the ANM study with serum lipid data available (first stage equation). To take into consideration the uncertainty in both the LOAD-GRS and the lipid trait-GRS associations we used the delta method to estimate the standard errors of the instrumental variable ratio estimates [30]. The resulting estimates were pooled together using inverse-variance fixed effects meta-analysis. We acknowledge that our instrumental variable (lipid trait-GRS associations) is calculated using only 550 participants from the ANM study. A recent study [31] has demonstrated that generating exposure data (such as lipid trait-GRS data) for a subset of participants rather than all participants in the study or from participants who are obtained from independent non-overlapping samples drawn from the same population does not substantially decrease power when the instrumental variable is relatively strong. An instrumental variable is considered strong when the first stage equation R^2^≈2.5% and, as demonstrated by Pierce and Burgess [31], full power is achieved for an R^2^≥1.5% with exposure data for ∼20% of the total sample. Additionally, loss in power is very small when the subsample/independent sample is 5%–10% of the total study sample. In our study groups, exposure data for the lipid trait-GRS association is available for 6% of the sample size of the MRC-WTCCC2 group (independent sample), for 83% of the IOP+ group (sub-sample), and for more participants than those in the ADNI group (independent sample). We have therefore used the instrumental variable estimate when the first stage equation for the four traits was R^2^≥1.5%.

#### Instrumental variable analysis using summary data

A second instrumental variable approach was used in order to verify findings of the instrumental variable approach using individual level data and to be used alternatively when the lipid trait-GRS first stage association was R^2^<1.5%. This approach was based on calculating the instrumental variable estimate using summary data approach, which has been shown to be similarly efficient to individual level data analyses [32]. Logistic regression analyses were used to test for the association of each lipid trait SNP with LOAD separately in each group adjusting for covariates as detailed above. As in the case of the GRS, we selected as the “risk” allele that which was associated with increased LDL-c, TG, and TC levels and decreased HDL-c levels. The instrumental variable estimate from summary data for each lipid trait was then obtained by summing the log OR of the individual logistic regression analyses of all SNPs associated with each plasma lipid trait and weighing this with the summary of the estimates of each SNP with the respective trait obtained from Willer and colleagues [20] in an inverse-variance weighted meta-analysis. The delta method was used to approximate the standard error [32].

Instrumental variable analysis was conducted using both full and trait specific scores.

### Additional Analyses

Additional analyses were performed excluding the population controls from the MRC-WTCCC2 group and adjusting for age at baseline visit, gender, and number of APOE ε4 alleles (Text S1).

## Results

The sample characteristics are presented in Table 1. SNPs details, including their minor allele frequency and association with LOAD in each group, are presented in Table S1.

**Table 1:** Characteristics of the participants in the MRC-WTCCC2, the IOP+, and ADNI study groups who passed GWA and imputation QC, broken down by cohort and by disease status.

Group	MRC-WTCCC2				IOP+			ADNI
Cohort	Total	MRC	WTCCC2 58BC	WTCCC2 NBS	Total	ANM	DCR	ADNI
		Illumina 610	Illumina 1.2M		Illumina 610			
**LOAD cases**								
*n*	3,234	3,234	N/A	N/A	350	285	65	330
Percent female	64	64	N/A	N/A	61	65	48	57
Mean age at onset (SD)	73 (9)	73 (9)	N/A	N/A	73 (7)	73 (7)	75 (7)	NA
Mean age at baseline (SD)	80 (8)	80 (8)	N/A	N/A	76 (7)	76 (7)	76 (7)	75 (7)
Mean age at death (SD)^a^	84 (8)	84 (8)	N/A	N/A	NA	NA	NA	NA
Mean HDL-c (SD) mg/dl^b^	N/A	N/A	N/A	N/A	63.1 (16)	63.1 (16)	NA	NA
Mean LDL-c (SD) mg/dl^b^	N/A	N/A	N/A	N/A	127.0 (37)	127.0 (37)	NA	NA
Mean TG (SD) mg/dl^b^	N/A	N/A	N/A	N/A	126.2 (48)	126.2 (48)	NA	NA
Mean TC (SD) mg/dl^b^	N/A	N/A	N/A	N/A	215.1 (43)	215.1 (43)	NA	NA
**Controls**								
*n*	6,164	1,175	2,602^c^	2,387^c^	313	226	87	187
Percent female	52	62	49	51	60	56	68	54
Mean age at baseline (SD)	63 (6)	77 (7)	60 (0)	N/A	74 (7)	73 (7)	76 (6)	76 (5)
Mean Age at death (SD)^a^	84 (8)	84 (8)	N/A	N/A	NA	NA	NA	NA
Mean HDL-c (SD) mg/dl^b^	N/A	N/A	N/A	N/A	60.9 (16)	60.9 (16)	NA	NA
Mean LDL-c (SD) mg/dl^b^	N/A	N/A	N/A	N/A	123.3 (33)	123.3 (33)	NA	NA
Mean TG (SD) mg/dl^b^	N/A	N/A	N/A	N/A	136.2 (59)	136.2 (59)	NA	NA
Mean TC (SD) mg/dl^b^	N/A	N/A	N/A	N/A	211.1 (41)	211.1 (41)	NA	NA

a Available only for 603 MRC LOAD cases and 101 LOAD controls.

b Available for 227 ANM LOAD cases and 196 ANM elderly controls; serum lipid levels (mmol/l) were converted to mg/dl by multiplying HDL-C, LDL-C, and TC by 38.67 and TG by 88.57.

c Population controls.

### Association of Blood Lipid Levels with the GRS and with LOAD in the ANM Cohort

The four full scores strongly correlated with the corresponding lipid trait phenotype; however, we observed no correlation between the trait specific scores and the corresponding lipid traits (Table 2) in the ANM individuals. We additionally observed no association between measured lipid levels and LOAD (Table 3) for this cohort.

**Table 2:** Association of the four full and trait specific GRSs with the respective serum levels in participants of the ANM cohort.

Trait	GRS (*n* SNPs)	ANM (*n* = 550) Respective Serum Lipid				
		beta	95% CI	*p*-Value	R^2^	F (1,548)
HDL-C	Full (69 SNPs)	0.210	0.12–0.30	3.14E−06	4.19%	22.2
	Trait specific (45 SNPs)	−0.016	−0.11 to 0.07	7.30E−01	0.02%	0.1
LDL-C	Full (55 SNPs)	0.136	0.05–0.22	2.19E−03	1.83%	9.5
	Trait specific (9 SNPs)	−0.051	−0.14 to 0.04	2.45E−01	0.28%	1.4
TG	Full (40 SNPs)	0.208	0.12–0.29	2.08E−06	4.34%	23.1
	Trait specific (16 SNPs)	0.072	−0.02 to 0.16	1.15E−01	0.50%	2.5
TC	Full (70 SNPs)	0.191	0.10–0.28	1.67E−05	3.59%	18.9
	Trait specific (18 SNPs)	0.054	−0.03 to 0.14	2.16E−01	0.31%	1.5

beta represents the association of each GRS with 1 unit increase in blood lipid levels. These associations include 127 MCI individuals.

**Table 3:** Association of serum lipid levels with LOAD in participants of the ANM cohort.

Serum Lipid Levels	ANM (*n* = 423)		
	OR	95% CI	*p*-Value
HDL-C	1.045	0.86–1.28	0.659
LDL-C	1.105	0.90–1.35	0.324
TG	0.870	0.71–1.07	0.182
TC	1.071	0.88–1.31	0.505

OR represents the association of 1 unit of each serum lipid with LOAD status.

### Expected Effect Size and Power of the Study

Using two epidemiological studies that have shown positive associations between cholesterol levels and LOAD and the observed associations between the weighted TC GRS and TC levels in the ANM sample, we estimated the expected effect sizes (Figure 1) and the power of our study. According to Whitmer and colleagues [7] midlife high TC levels (≥240 mg/dl) were associated with increased LOAD risk, hazard ratio [HR] = 1.42 (95% CI 1.22–1.66), after adjusting for other cardiovascular risk factors and, according to Kivipelto and colleagues [8], high TC levels (≥6.5 mmol/l, i.e., 251.35 mg/dl) were associated with increased LOAD risk, OR = 2.8 (95% CI 1.2–6.7) (Figure 1b). We dichotomised serum TC levels in the ANM subset according to these two studies and estimated their association with the TC GRS. The OR of high TC levels in our sample using the Whitmer and colleagues [7] cut-off (≥6.2 mmol/l) was OR = 1.679 (95% CI 1.35–2.10, *p* = 4.01×10^−6^) and the OR of high TC levels using Kivipelto and colleagues [8] cut-off (≥6.5 mmol/l) was OR = 1.71 (95% CI 1.34–2.20, *p* = 1.92×10^−5^) per GRS SD (Figure 1a) for the full GRS. This means that, if the weighted TC GRS is associated with LOAD through its association with TC levels we would expect the association of the GRS with LOAD (Figure 1c) to be between OR = 1.19 (95% CI 1.06–1.46) and OR = 1.73 (95% CI 1.06–4.48). Our sample size of >10,000 participants and the GRS approach we employed gave us >99% power to capture these ORs.

### Association of the Genotype Risk Scores with LOAD Status

**Figure 2:**
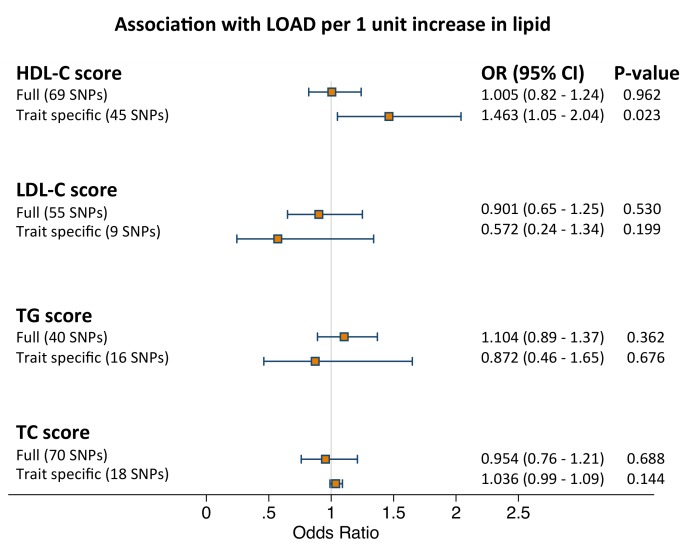
Results of the meta-analysis pooled estimates for the effect of a 1 unit increase in blood lipid traits on LOAD risk using instrumental variable analysis (*n* = 10,578). GRSs were calculated using all independent SNPs associated with each trait (full score) and SNPs associated exclusively with each trait (trait specific score). Full score estimates were derived by weighing the association between GRS and LOAD for each dataset with the association between GRS and blood lipid trait and pooling them together using inverse-variance fixed effects meta-analysis and by using the summary method. Restricted score estimates were derived by using the summary method since the trait specific score and blood lipid estimate was a weak instrument. See Methods for further details.

Instrumental variable analysis for the four full GRSs was performed using individual level data since the association of the four full GRSs with the corresponding lipid produced R^2^>0.015 (first stage equation). We found no association between any lipid traits and LOAD status (Figure 2; Table S2). Instrumental variable results for the four full GRSs using summary data based on the plasma lipid study by Willer and colleagues [20] were identical (Figure S2; Table S2).

Since we found no association between the four trait specific GRSs and the respective serum lipid (Table 2), instrumental variable analysis for the trait specific scores was performed using summary data only from Willer et al [20]. We observed a weak positive association between HDL-c and LOAD status when using the trait specific score and no associations for the rest of the traits (Figure 2; Table S2). Results of the logistic regression analyses for each of the four full and trait specific GRSs against LOAD status (second stage equations) are presented in Table 4.

**Table 4:** Association of lipid genotype risk scores with LOAD per lipid score SD using individual level data (stage 1 equation).

Trait	GRS (*n* SNPs)	MRC-WTCCC2 (*n* = 9,398^a^)			IOP+ (*n* = 663^a^)			ADNI (*n* = 517^a^)			MRC-WTCCC2, IOP+, and ADNI Meta-analysis (*n* = 10,578^a^)			
		OR	95% CI	*p*-Value	OR	95% CI	*p*-Value	OR	95% CI	*p*-Value	OR	95% CI	*p*-Value	Percent I^2^
HDL-C	Full (69 SNPs)	0.989	0.94–1.04	0.631	1.098	0.93–1.29	0.262	1.091	0.90–1.33	0.387	1.001	0.96–1.05	0.962	10
	Trait specific (45 SNPs)	1.047	1.00–1.10	0.060	1.133	0.96–1.34	0.149	0.975	0.80–1.19	0.807	1.049	1.00–1.10	0.036	0
LDL-C	Full (55 SNPs)	0.971	0.93–1.02	0.223	1.037	0.88–1.22	0.664	1.193	0.98–1.45	0.078	0.986	0.94–1.03	0.530	55
	Trait specific (9 SNPs)	0.958	0.91–1.00	0.074	0.961	0.81–1.14	0.642	1.180	0.97–1.44	0.104	0.968	0.93–1.01	0.150	50
TG	Full (40 SNPs)	1.025	0.98–1.07	0.305	0.905	0.77–1.07	0.234	1.129	0.93–1.38	0.226	1.021	0.98–1.07	0.362	35
	Trait specific (16 SNPs)	0.981	0.94–1.02	0.420	1.021	0.87–1.20	0.808	1.079	0.88–1.32	0.464	0.988	0.95–1.03	0.597	0
TC	Full (70 SNPs)	0.982	0.94–1.03	0.460	0.992	0.84–1.18	0.929	1.149	0.95–1.40	0.164	0.991	0.95–1.04	0.688	14
	Trait specific (18 SNPs)	1.034	0.99–1.09	0.165	0.961	0.81–1.14	0.644	0.809	0.65–1.00	0.050	1.018	0.97–1.06	0.442	63

Scores were calculated using all independent SNPs associated with each trait (full) and SNPs associated exclusively with each trait (trait specific) for all datasets and pooled together using inverse-variance fixed effects meta-analysis. Since there was some evidence for between study heterogeneity, random effects models were also tested but did not affect the meta-analysis results.

a Maximum.

Excluding population controls and adjusting for covariates produced similar results for all analyses (Tables 5 and 6).

**Table 5:** Association of lipid genotype risk scores with LOAD per one unit increase in lipid levels excluding population controls from the MRC-WTCCC2 group.

Trait	GRS (*n* SNPs)	Score Calculation Method	MRC (*n* = 4,409a)			IOP+ (*n* = 663^a^)			ADNI (*n* = 517^a^)			MRC, IOP+, and ADNI Meta-analysis (*n* = 5,589^a^)			
			OR	95% CI	*p*-Value	OR	95% CI	*p*-Value	OR	95% CI	*p*-Value	OR	95% CI	*p*-Value	Percent I^2^
HDL-C	Full (69 SNPs)	Individual level data	0.987	0.70–1.40	0.941	1.559	0.39–2.56	0.265	1.514	0.39–2.55	0.394	1.104	0.82–1.49	0.517	0
	Full (69 SNPs)	Summary data	0.992	0.75–1.31	0.959	1.411	0.76–2.62	0.280	1.399	0.66–2.95	0.384	1.085	0.85–1.38	0.508	0
	Trait specific (45 SNPs)		1.299	0.74–2.27	0.365	2.744	0.80–9.42	0.109	0.840	0.19–3.66	0.827	1.388	0.86–2.24	0.18	0
LDL-C	Full (55 SNPs)	Individual level data	0.623	0.36–1.07	0.088	1.309	0.24–4.22	0.677	3.652	0.24–4.22	0.077	0.840	0.53–1.34	0.465	65
	Full (55 SNPs)	Summary data	0.773	0.57–1.05	0.094	1.145	0.60–2.22	0.701	2.081	0.94–4.60	0.069	0.913	0.70–1.18	0.491	65
	Trait specific (9 SNPs)		0.840	0.20–3.45	0.82	0.401	0.02–11.07	0.604	27.220	0.60–1242	0.090	1.089	0.32–3.73	0.892	38
TG	Full (40 SNPs)	Individual level data	0.910	0.64–1.29	0.600	0.619	0.39–2.58	0.236	1.795	0.39–2.58	0.228	0.992	0.68–1.25	0.600	31
	Full (40 SNPs)	Summary data	0.910	0.64–1.29	0.607	0.632	0.30–1.34	0.235	1.74	0.71–4.29	0.231	0.923	0.69–1.24	0.595	30
	Trait specific (16 SNPs)		0.839	0.29–2.44	0.761	1.395	0.13–14.84	0.795	3.194	0.187–54.4	0.430	1.043	0.42–2.61	0.928	0
TC	Full (70 SNPs)	Individual level data	0.768	0.52–1.13	0.181	0.961	0.36–2.78	0.935	2.066	0.35–2.78	0.165	0.882	0.63–1.23	0.451	37
	Full (79 SNPs)	Summary data	0.823	0.61–1.10	0.195	0.971	0.51–1.85	0.936	1.791	083–3.88	0.141	0.917	0.71–1.18	0.501	41
	Trait specific (18 SNPs)		2.301	0.86–6.20	0.099	0.800	0.10–7.26	0.853	0.127	0.01–1.78	0.126	1.035	0.99–1.09	0.151	60

Scores were calculated using all independent SNPs associated with each trait (full) and SNPs associated exclusively with each trait (trait specific) for all datasets and pooled together using inverse-variance fixed effects meta-analysis. Full allele scores were calculated using both raw genotype data and summary data. Trait specific allele scores were calculated using summary data only. Since there was some evidence for between study heterogeneity, random effects models were also tested but did not affect the meta-analysis results.

a Maximum.

**Table 6:** Association of lipid genotype risk scores with LOAD per one unit increase in lipid levels after controlling for age at baseline visit, number of APOE e4 alleles, and gender.

Trait	GRS (*n* SNPs)	Score Calculation Method	MRC-WTCCC2 (*n* = 9,398^a^)			IOP+ (*n* = 663*)			ADNI (*n* = 517*)			MRC-WTCCC2, IOP+, and ADNI Meta-analysis (*n* = 10,578*)			
			OR	95% CI	*p*-Value	OR	95% CI	*p*-Value	OR	95% CI	*p*-Value	OR	95% CI	*p*-Value	Percent I^a^
HDL-C	Full (69 SNPs)	Individual level data	0.855	0.59–1.25	0.418	3.298	1.28–8.50	0.014	1.533	0.54–4.32	0.419	1.073	0.77–1.45	0.678	70
	Full (69 SNPs)	Summary data	0.882	0.65–1.20	0.426	2.454	1.17–5.16	0.018	1.408	0.62–3.21	0.422	1.056	0.81–1.38	0.690	70
	Trait specific (45 SNPs)		1.069	0.57–1.95	0.839	2.070	0.47–11.00	0.343	1.081	0.21–5.47	0.931	1.163	0.69–1.97	0.574	0
LDL-C	Full (55 SNPs)	Individual level data	0.630	0.35–1.34	0.124	0.801	0.18–3.52	0.769	2.818	0.58–13.61	0.193	0.762	0.46–1.28	0.302	35
	Full (55 SNPs)	Summary data	0.783	0.57–1.08	0.138	1.834	0.39–1.97	0.766	1.834	0.76–4.43	0.179	0.868	0.65–1.15	0.328	65
	Trait specific (9 SNPs)		0.540	0.12–2.47	0.435	0.020	0.001–1.05	0.060	16.270	0.22–1189	0.204	0.516	0.14–1.99	0.337	60
TG	Full (40 SNPs)	Individual level data	0.981	0.67–1.45	0.922	0.430	0.16–1.13	0.086	2.085	0.72–6.08	0.178	0.955	0.68–1.34	0.790	55
	Full (40 SNPs)	Summary data	0.981	0.67–1.42	0.928	0.454	0.18–1.12	0.088	1.939	0.71–5.30	0.198	0.954	0.69–1.32	0.595	50
	Trait specific (16 SNPs)		0.757	0.24–2.44	0.650	1.000	0.06–17.1	0.999	7.990	0.343–185	0.197	1.001	0.36–2.76	0.998	0
TC	Full (70 SNPs)	Individual level data	0.733	0.48–1.12	0.148	1.038	0.35–3.45	0.946	1.708	0.55–5.34	0.357	0.835	0.58–1.21	0.340	14
	Full (79 SNPs)	Summary data	0.798	0.58–1.09	0.161	1.010	0.46–2.21	0.983	1.541	0.65–3.65	0.330	0.880	0.67–1.16	0.364	5
	Trait specific (18 SNPs)		1.609	0.55–4.69	0.390	0.275	0.02–3.85	0.342	0.090	0.01–1.60	0.100	0.951	0.37–2.43	0.917	55

Age for the WTCCC2 population controls was set to 60 years (See Methods). Scores were calculated using all independent SNPs associated with each trait (full) and SNPs associated exclusively with each trait (trait specific) for all datasets and pooled together using inverse-variance fixed effects meta-analysis. Full allele scores were calculated using both raw genotype data and summary data. Trait specific allele scores were calculated using summary data only. Since there was some evidence for between study heterogeneity, random effects models were also tested but did not affect the meta-analysis results.

a Maximum.

## Discussion

The aim of this study was to dissect the causal nature of the association between blood lipid levels and LOAD and to investigate whether genetic predisposition to dyslipidemia plays an aetiological role in LOAD. To achieve this we used a Mendelian randomization approach and we examined the causal role of HDL-c, LDL-c, TG, and TC in LOAD risk by exploring the association of GRS based on the additive joint effect of 157 well established genetic loci [20] that influence plasma HDL-c, LDL-c, TC, and TG levels with LOAD in a sample of >10,000 participants. Full GRSs were constructed including all SNPs associated with the respective trait at *p*<5×10^−8^. Instrumental variable analysis took place using individual level data and calculating the instrumental variable estimate by dividing the LOAD-GRS estimate for each lipid trait with the respective lipid trait-GRS estimate for each study and pooling them together using meta-analysis, when the association of the lipid trait-GRS was R^2^>1.5%. Instrumental variable analysis using summary data was used to verify individual level results and when the lipid trait-GRS was R^2^<1.5%. Since one of the prerequisites for a Mendelian randomization study is that there must not be pleiotropic effects of the genetic variants of interest, we additionally attempted to dissect these associations further and we constructed trait specific GRSs including SNPs associated exclusively with each lipid phenotype.

We found no association between any of the full GRSs and LOAD risk. Our results suggest that genetically raised HDL-c, LDL-c, TG, and TC levels are not causally associated with LOAD risk. Results for the trait specific scores were similar. Although we observed a positive association between the HDL-C GRS and LOAD we must acknowledge the large standard error of the association and that the trait specific score is a weaker instrument. The 157 loci account approximately for 12%–14% of the variation of each trait [20]; however, there are no published results of the trait variance explained by SNPs exclusively associated with each trait. In our study we observed a clear difference between the full GRS (R^2^ = 1.8%–4.3%) and the trait specific GRS (R^2^≤0.5%) highlighting that although the trait specific score had increased specificity for the target lipid, it is less statistically powerful and consequently less biologically interpretable. Finally, excluding population controls and adjusting for covariates produced similar results.

### Clinical Relevance

Altered lipid metabolism has been extensively implicated in AD pathogenesis through cell biological, epidemiological, and genetic studies, but the molecular mechanisms linking cholesterol and risk for AD are still not well understood. This relationship between AD and altered lipid metabolism is of considerable interest for both basic scientists and clinicians. This is the first study, to our knowledge, to model the joint additive effect of lipid associated loci on LOAD risk using a Mendelian randomization approach. Therefore this article contributes considerably to research on the role of lipids in risk for LOAD and has potential for suggesting novel therapeutic and public health interventions.

### Strengths and Limitations

One of the strengths of this study is that genetic variants combined into a GRS to test for a complex association between metabolic traits and LOAD in a very large sample using Mendelian randomization were used. The additive effects of these SNPs have been found to be highly associated with the extremes of the distribution for each trait. For example, in a meta-analysis by Teslovich and colleagues [33] using a smaller number of lipid associated loci (*n* = 95), the OR of high plasma LDL-c (mean 219 mg/dl) against low plasma LDL-c (mean 110 (mg/dl) for individuals on the top LDL-c GRS quartile was 12.5 (95% CI 9.1–17.5, *p* = 1×10^−14^). Here, in the subset with serum lipid levels we observed strong associations between all of the GRSs and the corresponding serum lipid levels, which are similar to those previously published for plasma lipid levels. Using two epidemiological studies that have shown positive associations between HR = 1.45 and OR = 2.8 (the majority of positive associations reported are OR/HR between 2 to 3) and the association between TC GRS and abnormally high serum TC levels in the ANM cohort, the expected effect sizes in this study were estimated to be between OR = 1.20 and OR = 1.70 per GRS SD giving us >99% power to capture these associations. Using individual level data and summary data when appropriate, we calculated instrumental variable estimators assessing the association of the GRS with LOAD status per increase in 1 lipid unit.

Mendelian randomization studies overcome biases found in non-genetic studies such as confounding and reverse causation. For example, epidemiological studies investigating the association of lipid levels and LOAD can be biased from confounding factors that may affect lipid levels, from the co-occurrence of other conditions that may be associated with LOAD such as impaired glucose metabolism and obesity, from the type of study performed, from the fact that the majority of the studies have only used total cholesterol measurements, from the different blood lipid cut-offs used for analysis (or their use as continuous variable), and, most importantly, from the different timing of lipid measurements in relation to age and disease onset. For example many studies have been conducted late in the life of the participant when substantial AD neuropathology may already be present and it is therefore difficult to determine whether any changes in cholesterol levels are increasing risk for disease or, conversely, whether the pathophysiological changes that accompany AD alter cholesterol levels (reviewed in [34]). Interestingly, it has been shown that total cholesterol decreases with age [35], which may reflect ongoing disease processes and it has been suggested that as blood pressure and body mass index have been shown to begin to decline several years before dementia, the same may occur for LDL-c levels, supporting a role for reverse causation. Indeed, for males in the Honolulu-Asia study comparing total cholesterol levels across 26 years, an accelerated pre-clinical decline in cholesterol was observed for those who subsequently developed AD [15]. A recent study examined serum cholesterol levels and cerebral Aβ measured with carbon C11-labeled Pittsburgh Compound B (PIB) and found a negative association between HDL-c and global PIB index and a positive association between LDL-c and global PIB index [36]. Although these findings are consistent with some epidemiological and clinical studies, the authors acknowledge that the measurements are cross-sectional, obtained late in life, and from a high vascular risk population; hence these associations could be confounded through reverse causation as other cross sectional studies. Genetic studies, however, overcome these issues since they are more likely to reflect lifelong exposure to altered circulating blood lipid levels.

Our study, similar to other Mendelian randomization studies, suffers from potential limitations [37]–[39], which are related to the validity of the assumptions underlying these studies. The main assumptions are: (a) independence between instrument and covariates, i.e., that the tested genotypes in the GRS are randomized; (b) a reliable association between the GRS and the intermediate phenotype (first stage equation), and (c) lack of pleiotropy. Although we know that there is no violation of the second assumption particularly in the case of the full GRS, possible violations of the first and third assumptions include population stratification, pleiotropic effects, canalization, epigenetic effects, and the confounding effect of genes associated with confounders and outcomes in high linkage disequilibrium with genotypes. Population stratification is not present in the current study since a white population has been used and allele frequencies between the different cohorts are very similar. Additionally, pleiotropy and the potentially confounding effects of linkage disequilibrium are likely avoided owing to the use of multiple genetic variants in the GRS and the use of the trait specific scores. Nevertheless, canalization cannot be completely excluded as a limitation of the present study.

Another limitation of our study, linked also to Mendelian randomization assumption two, is that that serum lipid levels were only available for a small proportion of the sample (227 LOAD cases, 196 controls and 127 MCI from the ANM cohort). We used the 550 participants from ANM dataset to calculate the GRS-lipid trait association (first stage equation) for the individual level data instrumental variable analysis in order to derive these estimates from a sample nested within our total sample. We also used the 550 participants from the ANM cohort in order to infer the expected association between the GRS with LOAD in our power analysis, since we could not find any available large published studies where the same cut-off for cholesterol levels was used when investigating its association with GRS and with LOAD. We have to additionally acknowledge that since these estimates are derived from the ANM cohort they may not apply to the other cohorts.

Although we acknowledge the small number of participants, all the full GRSs were strongly associated with the respective lipid with R^2^ = 1.8%–4.3% (strong instruments) and it is shown [31] that full power is achieved for an R^2^>1.5% with exposure data for ∼20% of the total sample/independent sample and with very small loss in power when the subsample/independent sample is 5%–10% of the total study sample. Moreover, we acknowledge that our lipid measurements for the ANM cohort come from serum as opposed to the Willer and colleagues [20] study which was based on plasma lipid measurements. Consequently, the association between the GRS and lipids and the instrumental variable analysis using individual level data are based on lipid serum data. On the other hand, our instrumental variable analysis using summary data is based on plasma lipid data from Willer and colleagues [20]. The association between the GRS and serum lipid levels in our sample closely reflect those for plasma in published studies (for example, Teslovich and colleagues [33]). Additionally, the suitability of our instruments when R^2^>1.5% was verified by performing instrumental variable analysis using summary data.

Another limitation was that although no association was observed between the GRS and age in cases or controls suggesting no survival effect, cases were on average older than controls. We also included ∼6,000 population controls, ∼3,000 of whom were <60 years and could therefore develop AD in the future, and ∼3,000 of this group had no age or cognitive level information. Additionally, samples from the WTCCC2 1958 Birth Cohort were also included in the Global Lipids Consortium study. However, when we repeated analyses excluding the population controls, results were essentially identical. A strength of our large case control study is that diagnosis of AD is standardised and performed under a research setting and for a proportion of cases and cognitively normal elderly controls diagnosis was confirmed by pathological examination. We must also take into consideration that case-control studies have the potential for selection or ascertainment biases in the inclusion of cases with dyslipidemia associated problems (e.g., cardiovascular disease [CVD]). However, CVD co-morbidity was not excluded here, which would result in an artificially healthy case group with beneficial lipid-influencing genetic profiles. Information on history of myocardial infarction was available only for 643 MRC participants (521 cases and 132 controls).

### Generalizability

The relationship between lipid metabolism and LOAD is likely to be complex. The blood-brain-barrier prevents any efficient exchange between brain and blood lipoproteins, and the majority of brain cholesterol is derived from *de novo* biosynthesis, rather than from blood LDL-c [40]; cholesterol levels in the periphery may therefore not reflect brain cholesterol levels. Additionally, although excess free cholesterol in brain is metabolised into cholesteryl-esters, it is also converted into 24(S)-hydroxycholesterol, an oxidized metabolite of cholesterol, which crosses the blood-brain-barrier and reaches the periphery. It has been shown that during the early stages of AD, blood 24S-hydroxycholesterol concentrations, which reflect TC concentrations in the brain, are high in cerebrospinal fluid and in peripheral circulation, potentially reflecting increased cholesterol turnover in the brain but fall in later stages of AD suggesting a lower rate of cholesterol metabolism as disease progresses (reviewed in [41]).

### Conclusions

There is no dispute over the involvement of lipid metabolism in the pathophysiology of LOAD. However, the results of our study do not support a causal role for genetically increased plasma cholesterol in LOAD and suggest that epidemiological associations between peripheral lipids and LOAD may be confounded by secondary disease processes. Future studies should focus on large LOAD datasets with longitudinal peripheral lipid measures and other markers of lipid metabolism.

## Supporting Information

Figure S1 **Venn diagram illustrating the overlap of SNPs associated with the four lipid traits used in this study.** The rs4420638 SNP in the APOE locus, the SNP rs581080 in the TTC39B locus, the SNP rs9411489 in the ABO locus, and the SNP rs3177928 in the HLA locus are excluded.
(TIF)

Figure S2 **Results of the meta-analysis pooled estimates for the effect of a 1unit increase in blood lipid traits on LOAD risk using instrumental variable analysis (full genotype risk scores), using the summary method (** ***n*** = 10.578*). Estimates were derived by weighing the association between GRS and LOAD for each dataset with the association between GRS and blood lipid using the summary method. See Methods for further details. *Maximum.
(TIF)

Table S1 **Information on SNPs used for the construction of the genotype risk scores in this study.** SNPs used for the four GRSs constructed in this study and their association with the respective plasma levels in Willer and colleagues [20], as well as their association with LOAD in the three groups in this study. Risk allele refers to the allele associated with increasing LDL-c, TC, and TG levels and decreasing HDL-c levels. Analyses are performed with respect to the risk allele. Risk allele frequency refers to the frequency of the risk allele used in the analysis. For the four full GRSs all SNPs associated with the specific phenotype at *p*<5×10^−8^ were used. For the trait specific GRSs only SNPs associated exclusively with each lipid trait at *p*<5×10^−8^ were used. The APOE rs4420638 SNP was not included in the calculation of the GRS because of its association with LOAD. rs581080 in TTC39B locus, rs9411489 of ABO locus, and rs3177928 in HLA locus were not successfully genotyped/imputed and we found no SNPs with R^2^>0.8 that were successfully genotyped/imputed to be used as proxies. *Maximum.
(XLSX)

Table S2 **Association of lipid genotype risk scores with LOAD per one unit increase in lipid levels.**
(DOCX)

Text S1 **Extended methods.**
(DOCX)

## Abbreviations

AD: Alzheimer disease
ANM: AddNeuroMed
APOE: apolipoprotein E
GRS: genotype risk score
GWA: genome wide association
HDL-c: high-density lipoprotein cholesterol
IOP: Institute of Psychiatry
LDL-c: low-density lipoprotein cholesterol
LOAD: late onset Alzheimer disease
MCI: mild cognitive impairment
MRC: Medical Research Council
NBS: National Blood Donors
OR: odds ratio
QC: quality control
SD: standard deviation
TC: total cholesterol
TG: triglycerides
WTCCC2: Wellcome Trust Case Control Consortium
