# Supplementary figures and images for: Genetic Predisposition to Increased Blood Cholesterol and Triglyceride Lipid Levels and Risk of Alzheimer Disease: A Mendelian Randomization Analysis
Source: PLoS Med. 2014 Sep 16;11(9):e1001713. doi: 10.1371/journal.pmed.1001713 (PMC4165594; doi:10.1371/journal.pmed.1001713)

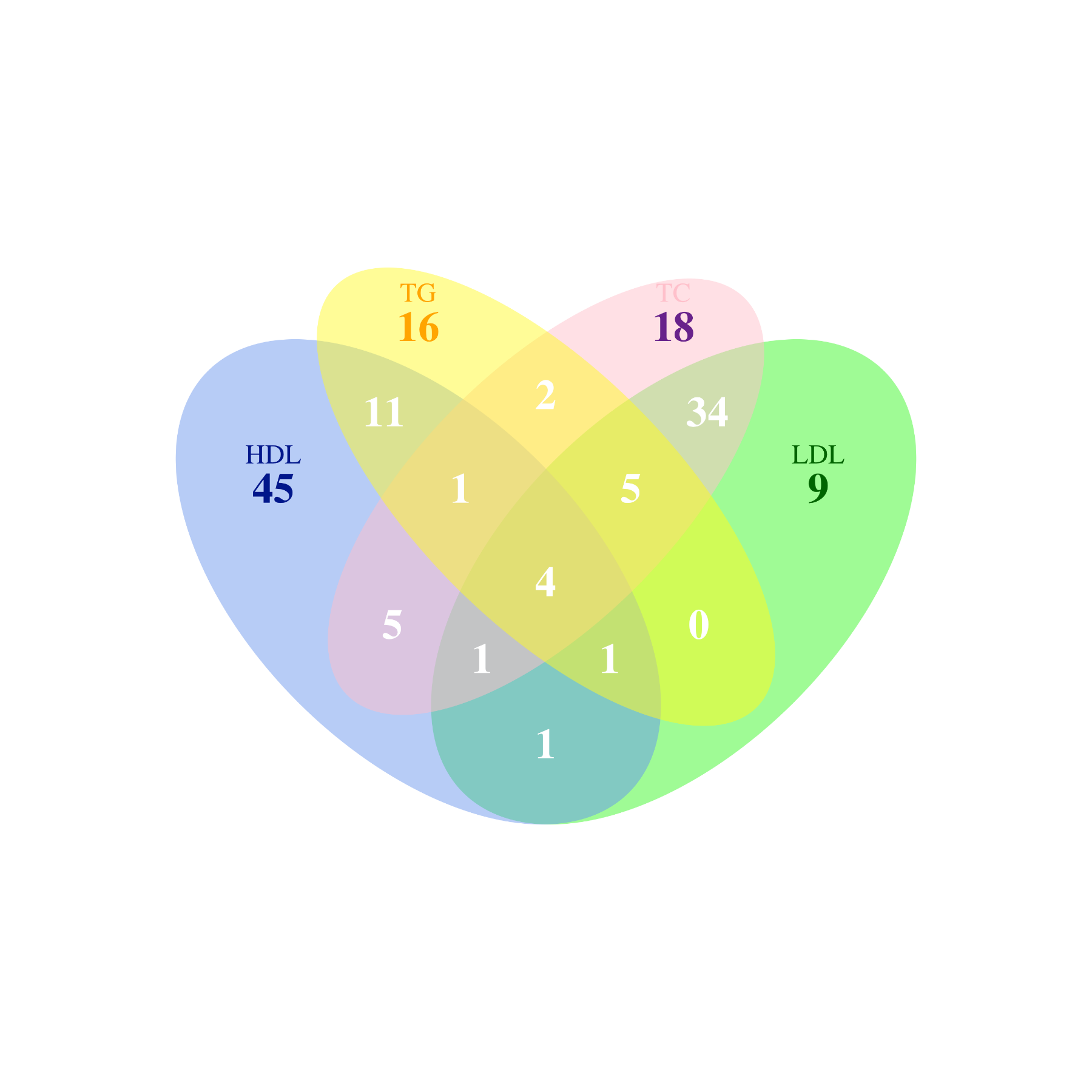

Supplement: Figure S1 — Venn diagram illustrating the overlap of SNPs associated with the four lipid traits used in this study. The rs4420638 SNP in the APOE locus, the SNP rs581080 in the TTC39B locus, the SNP rs9411489 in the ABO locus, and the SNP rs3177928 in the HLA locus are excluded. (TIF) [file pmed.1001713.s001.tif]

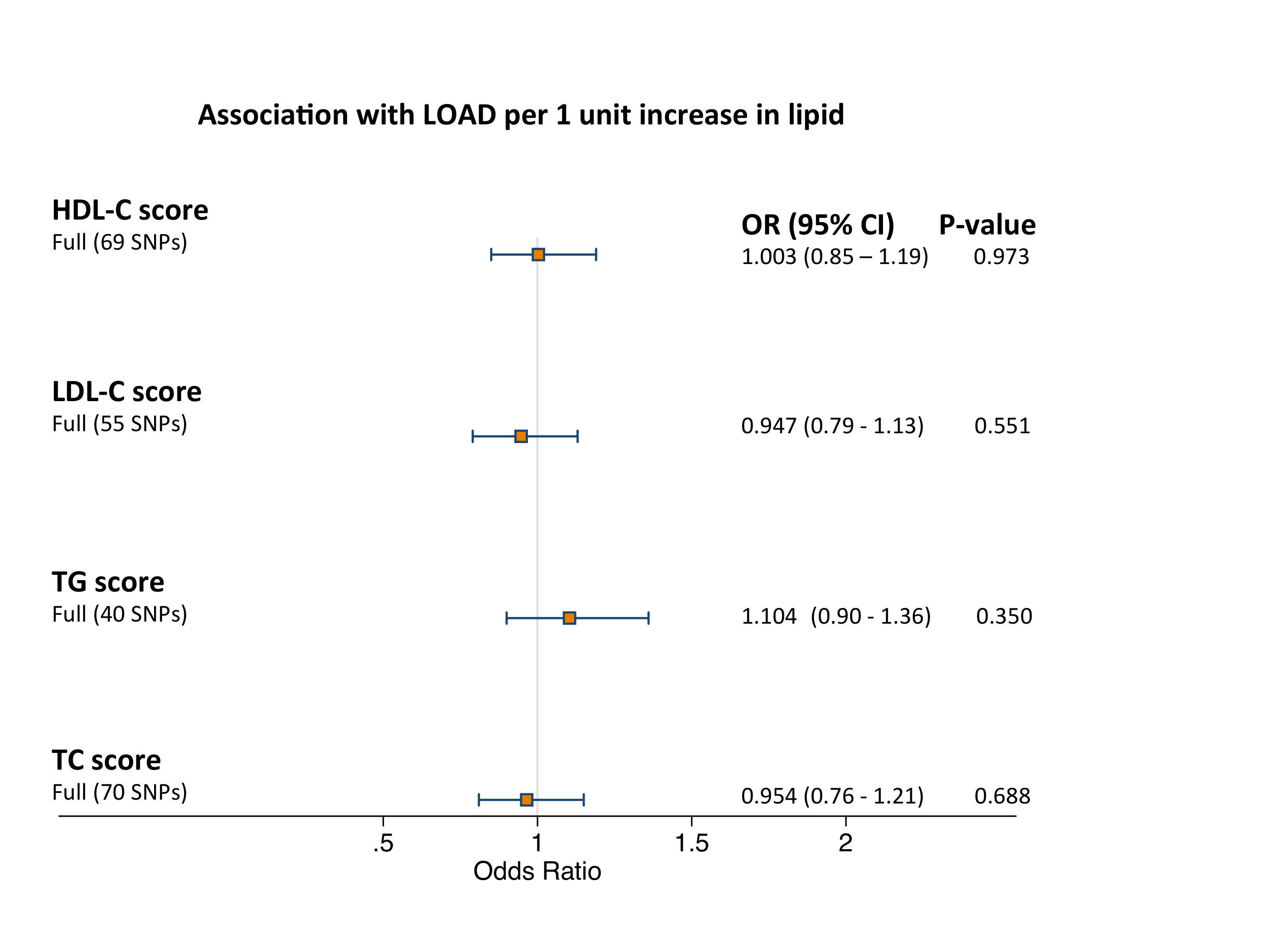

Supplement: Figure S2 — Results of the meta-analysis pooled estimates for the effect of a 1unit increase in blood lipid traits on LOAD risk using instrumental variable analysis (full genotype risk scores), using the summary method ( n = 10.578*). Estimates were derived by weighing the association between GRS and LOAD for each dataset with the association between GRS and blood lipid using the summary method. See Methods for further details. *Maximum. (TIF) [file pmed.1001713.s002.tif]
