# Supplementary material for: Genetic Predisposition to Increased Blood Cholesterol and Triglyceride Lipid Levels and Risk of Alzheimer Disease: A Mendelian Randomization Analysis
Source: PLoS Med. 2014 Sep 16;11(9):e1001713. doi: 10.1371/journal.pmed.1001713 (PMC4165594; doi:10.1371/journal.pmed.1001713)
Supplement: Table S2 — Association of lipid genotype risk scores with LOAD per one unit increase in lipid levels. (DOCX) [file pmed.1001713.s004.docx]

Table S3. Association of lipid Genotype Risk Scores with LOAD per one unit increase in lipid levels.

| **Trait** | **Genotype Risk Score**  **(N SNPs)** | **Score calculation method** | **MRC-WTCCC2 (N=9398*)** | | | **IOP +**  **(N=663*)** | | | **ADNI**  **(N=517*)** | | | **MRC-WTCCC2, IOP+ &ADNI meta- analysis (N=10578*)** | | | |
| --- | --- | --- | --- | --- | --- | --- | --- | --- | --- | --- | --- | --- | --- | --- | --- |
|  |  |  |  |  |  |  |  |  |  |  |  |  |  |  |  |
|  |  |  |  |  |  |  |  |  |  |  |  |  |  |  |  |
|  |  |  | **OR** | **95%CI** | **p** | **OR** | **95%CI** | **p** | **OR** | **95%CI** | **p** | **OR** | **95%CI** | **p** | **Percent I^2^** |
| HDL-C | Full (69 SNPs) | Individual level data | 0.947 | 0.76 - 1.18 | 0.644 | 1.559 | 0.39 - 2.56 | 0.265 | 1.514 | 0.39 - 2.55 | 0.394 | 1.005 | 0.82 - 1.24 | 0.962 | 10 |
|  | Full (69 SNPs) | Summary data | 0.956 | 0.80 - 1.14 | 0.635 | 1.411 | 0.76 - 2.62 | 0.280 | 1.399 | 0.66 - 2.95 | 0.384 | 1.003 | 0.85 - 1.19 | 0.973 | 9 |
|  | Trait specific (45 SNPs) |  | 1.434 | 1.00 - 2.05 | 0.048 | 2.744 | 0.80 - 9.42 | 0.109 | 0.840 | 0.19 - 3.66 | 0.827 | 1.463 | 1.05 - 2.04 | 0.023 | 0 |
| LDL-C | Full (55 SNPs) | Individual level data | 0.807 | 0.57 - 1.14 | 0.225 | 1.309 | 0.24 - 4.22 | 0.677 | 3.652 | 0.24 - 4.22 | 0.077 | 0.901 | 0.65 - 1.25 | 0.530 | 55 |
|  | Full (55 SNPs) | Summary data | 0.889 | 0.73 - 1.07 | 0.235 | 1.145 | 0.60 - 2.22 | 0.701 | 2.081 | 0.94 - 4.60 | 0.069 | 0.947 | 0.79 - 1.13 | 0.551 | 56 |
|  | Trait specific (9 SNPs) |  | 0.471 | 0.19 - 1.18 | 0.105 | 0.401 | 0.02 - 11.07 | 0.604 | 27.220 | 0.60 - 1242 | 0.090 | 0.572 | 0.24 - 1.34 | 0.199 | 50 |
| TG | Full (40 SNPs) | Individual level data | 1.126 | 0.90 - 1.41 | 0.309 | 0.619 | 0.39 - 2.58 | 0.236 | 1.795 | 0.39 - 2.58 | 0.228 | 1.104 | 0.89 - 1.37 | 0.362 | 35 |
|  | Full (40 SNPs) | Summary data | 1.127 | 0.90 - 1.41 | 0.295 | 0.632 | 0.30 - 1.34 | 0.235 | 1.74 | 0.71 - 4.29 | 0.231 | 1.104 | 0.90 - 1.36 | 0.350 | 36 |
|  | Trait specific (16 SNPs) |  | 0.778 | 0.39 - 1.54 | 0.481 | 1.395 | 0.13 - 14.84 | 0.795 | 3.194 | 0.187 - 54.4 | 0.43 | 0.872 | 0.46 - 1.65 | 0.676 | 36 |
| TC | Full (70 SNPs) | Individual level data | 0.911 | 0.71 - 1.17 | 0.469 | 0.961 | 0.36 - 2.78 | 0.935 | 2.066 | 0.35 - 2.78 | 0.165 | 0.954 | 0.76 - 1.21 | 0.688 | 14 |
|  | Full (79 SNPs) | Summary data | 0.931 | 0.77 - 1.12 | 0.461 | 0.971 | 0.51 - 1.85 | 0.936 | 1.791 | 083 - 3.88 | 0.141 | 0.965 | 0.81 - 1.15 | 0.694 | 23 |
|  | Trait specific (18 SNPs) |  | 1.577 | 0.83 - 2.97 | 0.163 | 0.800 | 0.10 - 7.26 | 0.853 | 0.127 | 0.01 - 1.78 | 0.126 | 1.036 | 0.99 - 1.09 | 0.144 | 50 |

Scores were calculated using all independent SNPs associated with each trait (Full) and SNPs associated exclusively with each trait (Trait specific) for all datasets and pooled together using inverse-variance fixed effects meta-analysis. Full allele scores were calculated using both raw genotype data and summary data. Trait specific allele scores were calculated using summary data only. Since there was some evidence for between study heterogeneity, random effects models were also tested but did not affect the meta-analysis results.
