# Supplementary material for: Genetic Predisposition to Increased Blood Cholesterol and Triglyceride Lipid Levels and Risk of Alzheimer Disease: A Mendelian Randomization Analysis
Source: PLoS Med. 2014 Sep 16;11(9):e1001713. doi: 10.1371/journal.pmed.1001713 (PMC4165594; doi:10.1371/journal.pmed.1001713)
Supplement: Text S1 — Extended methods. (DOCX) [file pmed.1001713.s005.docx]

### **TEXT S1. EXTENDED METHODS**

**1. STUDY SAMPLE**

**MRC-WTCCC2 group:** We used 4515 individuals (3292 AD cases and 1223 controls) from the GERAD1 (Genetic and Environmental Risk for Alzheimer’s disease) consortium (Harrold et al [1] GWA study) genotyped at the Sanger Institute on the Illumina 610-quad chip. These samples were recruited by the Medical Research Council (MRC) Genetic Resource for AD (Cardiff University; Kings College London; Cambridge University; Trinity College Dublin), the Alzheimer’s Research UK (ARUK) Collaboration (University of Nottingham; University of Manchester; University of Southampton; University of Bristol; Queen’s University Belfast; the Oxford Project to Investigate Memory and Ageing (OPTIMA), Oxford University); Washington University, St Louis, United States; MRC PRION Unit, University College London; London and the South East Region AD project (LASER-AD), University College London; Competence Network of Dementia (CND) and Department of Psychiatry, University of Bonn, Germany and the National Institute of Mental Health (NIMH) AD Genetics Initiative. AD [2].We also used 28 individuals (18 AD cases and 10 controls) from King’s College (MRC brain cohort) for whom brain tissue was available for DNA extraction and who were either part of the GERAD1 cohort and failed QC or who had not blood DNA available during the GERAD1 cohort genotyping. These were genotyped on the Illumina 666W-Quad chip.

Finally, 5,074 population controls were used from the Wellcome Trust Case Control Consortium 2 (WTCCC2: [www.wtccc.org.uk/ccc2/](http://www.wtccc.org.uk/ccc2/)). These comprised of 2,668 population controls from the 1958 British Birth Cohort (the 58BC cohort) and 2,406 from the UK Blood Service Collection (the NBS cohort), both cohorts genotyped on Illumina 1.2M chip.

**IOP+ group:** The Institute of Psychiatry Plus (IOP+) group (450 LOAD cases, 365 elderly controls) utilized individuals from the AddNeuroMed (ANM) cohort [3] (362 LOAD cases, 237 elderly controls) and individuals from the Dementia Case Register (88 LOAD cases and 128 elderly controls). These were genotyped on the Illumina 610-Quad chip in two different batches (Batch 1: 222 LOAD cases, 111 elderly controls; Batch 2: 228 LOAD cases and 254 controls). Additionally, Batch 1 contained 99 and Batch 2 contained 78 individuals with Mild Cognitive Impairment (MCI), a subset of which had serum lipid levels available.

**ADNI group:** The Alzheimer’s disease Neuroimaging Initiative (ADNI) group (330 LOAD cases, 187 elderly controls) utilized individuals from the Alzheimer’s Disease Neuroimaging Initiative (ADNI) database (adni.loni.usc.edu), genotyped on the Illumina 610-Quad chip.

The ADNI was launched in 2003 by the National Institute on Aging (NIA), the National Institute of Biomedical Imaging and Bioengineering (NIBIB), the Food and Drug Administration (FDA), private pharmaceutical companies and non-profit organizations, as a $60 million, 5- year public-private partnership. The primary goal of ADNI has been to test whether serial magnetic resonance imaging (MRI), positron emission tomography (PET), other biological markers, and clinical and neuropsychological assessment can be combined to measure the progression of mild cognitive impairment (MCI) and early Alzheimer’s disease (AD). Determination of sensitive and specific markers of very early AD progression is intended to aid researchers and clinicians to develop new treatments and monitor their effectiveness, as well as lessen the time and cost of clinical trials.

The Principal Investigator of this initiative is Michael W. Weiner, MD, VA Medical Center and University of California – San Francisco. ADNI is the result of efforts of many co-investigators from a broad range of academic institutions and private corporations, and subjects have been recruited from over 50 sites across the U.S. and Canada. The initial goal of ADNI was to recruit 800 subjects but ADNI has been followed by ADNI-GO and ADNI-2. To date these three protocols have recruited over 1500 adults, ages 55 to 90, to participate in the research, consisting of cognitively normal older individuals, people with early or late MCI, and people with early AD. The follow up duration of each group is specified in the protocols for ADNI-1, ADNI-2 and ADNI-GO. Subjects originally recruited for ADNI-1 and ADNI-GO had the option to be followed in ADNI-2. For up-to-date information, see [www.adni-info.org](http://www.adni-info.org).

**2. IMPUTATION**

Imputation took place using IMPUTE_2.2.2[4] and the 1000G phase1 integrated reference panel (April 2012, NCBI build 37). The imputation steps included: a) Data preparation (Pre-imputation QC has already taken place and symmetric SNPs were subsequently removed) and conversion of the 4 datasets to hg19 (NCBI build 37); b) Prephasing of each chromosome of each dataset using SHAPEIT[5]; c) Data division into 5Mb chunks and imputation of each chunk; d) Merging of imputed chunks and post-imputation QC (Data QC/filtering and conversion to used formats) using QCTOOL (www.well.ox.ac.uk/~gav/qctool) and the following criteria: Info score >0.4, MAF >=0.005, SNP missing rate < 0.05 and HWE thresholds of p<10^-10^ (after QQ plots inspection). For this project data were converted to PLINK format using GTOOL (http://www.well.ox.ac.uk/~cfreeman/software/gwas/gtool.html) and a probability threshold of >0.80. These resulted in >9 million SNPs, IN/DELs and SNVs for each dataset.

**3. ADDITIONAL ANALYSES**

Additional analyses were performed excluding the population controls from the MRC-WTCCC2 data since no age and cognitive function data were available for the WTCCC2 NBS cohort and WTCCC2 58BC participants were <54. Additionally the WTCCC2 58BC cohort was also included in the Global Lipids Consortium. Secondary models adjusting for age at baseline visit, gender and number of APOE ε4 alleles were also tested for association. For all datasets, rs429358 and rs7412 were imputed and the APOE haplotype was inferred. We observed <5% discordance in MRC and ANM samples having both genotyped and imputed data and the imputed APOE was therefore used for all individuals. Since no age was available for the WTCCC2 NBS cohort, all WTCCC2 ages were set at 60 i.e. the oldest age a subject can be assumed to be disease free before late onset AD symptoms can be diagnosed.

References

1. Harold D, Abraham R, Hollingworth P Sims R, Gerrish A et al. (2009) Genome-wide association study identifies variants at CLU and PICALM associated with Alzheimer's disease. Nat Genet 41: 1088-1093.
2. McKhann G, Drachman D, Folstein M, Katzman R, Price D et al. (1984) Clinical diagnosis of Alzheimer's disease: report of the NINCDS-ADRDA Work Group under the auspices of Department of Health and Human Services Task Force on Alzheimer's Disease. Neurology 34: 939-944.
3. Lovestone S, Francis P, Strandgaard K (2007) Biomarkers for disease modification trials--the innovative medicines initiative and AddNeuroMed. J Nutr Health Aging 11: 39-61.
4. Howie BN, Donnelly P, Marchini J (2009) A flexible and accurate genotype imputation method for the next generation of genome-wide association studies. PLoS Genet 5: e1000529.
5. Delaneau O, Marchini J, Zagury JF (2012) A linear complexity phasing method for thousands of genomes. Nat Methods 9: 179-181.
